# A Possible Role for Arylsulfatase G in Dermatan Sulfate Metabolism

**DOI:** 10.3390/ijms21144913

**Published:** 2020-07-12

**Authors:** Aleksandra Poterala-Hejmo, Adam Golda, Marcin Pacholczyk, Sebastian Student, Anna Tylki-Szymańska, Anna Lalik

**Affiliations:** 1 Department of Systems Biology and Engineering, Silesian University of Technology, 44-100 Gliwice, Poland; marcin.pacholczyk@polsl.pl (M.P.); sebastian.student@polsl.pl (S.S.); 2 Department of Cardiology, 4th Municipal Hospital, 44-100 Gliwice, Poland; adamgolda@interia.eu; 3 Department of Pediatrics, Nutrition and Metabolic Diseases, The Children’s Memorial Health Institute, 04-730 Warsaw, Poland; a.tylki@czd.pl

**Keywords:** arylsulfatase, dermatan sulfate, mucopolysaccharidosis, smooth muscle cell

## Abstract

Perturbations of glycosaminoglycan metabolism lead to mucopolysaccharidoses (MPS)—lysosomal storage diseases. One type of MPS (type VI) is associated with a deficiency of arylsulfatase B (ARSB), for which we previously established a cellular model using pulmonary artery endothelial cells with a silenced *ARSB* gene. Here, we explored the effects of silencing the *ARSB* gene on the growth of human pulmonary artery smooth muscle cells in the presence of different concentrations of dermatan sulfate (DS). The viability of pulmonary artery smooth muscle cells with a silenced *ARSB* gene was stimulated by the dermatan sulfate. In contrast, the growth of pulmonary artery endothelial cells was not affected. As shown by microarray analysis, the expression of the arylsulfatase G (*ARSG*) in pulmonary artery smooth muscle cells increased after silencing the arylsulfatase B gene, but the expression of genes encoding other enzymes involved in the degradation of dermatan sulfate did not. The active site of arylsulfatase G closely resembles that of arylsulfatase B, as shown by molecular modeling. Together, these results lead us to propose that arylsulfatase G can take part in DS degradation; therefore, it can affect the functioning of the cells with a silenced arylsulfatase B gene.

## 1. Introduction

Arylsulfatase catalyzes the hydrolysis of sulfate ester bonds (O-sulfatase activity) in glycosaminoglycans (GAG), sulfate esters, small aromatic molecules, and sulfolipids [1,2]. The human arylsulfatases family contains 11 members: A, B, C, D, E, F, G, H, I, J, and K. The human arylsulfatases A (ARSA) and B (ARSB) are present in lysosomes, arylsulfatase C (ARSC, STS) is a microsomal protein, and arylsulfatases D, F, H, J, and K are localized in ER membrane. In contrast, arylsulfatase E is present in the Golgi apparatus [3]. Arylsulfatase G is found in both ER and lysosomes [4,5]. Arylsulfatase I is found in the ER but may also be secreted [6]. All family members share 20–60% amino acid homology, reflected by similarities in their active site architecture and tertiary structures. Highly conserved motifs are present especially in the N-terminal part of the chain containing active site residues as well as binding sites for divalent ions, which suggests a similar mechanism of action. Their activity is dependent on the presence of a cysteine posttranslationally modified to formylglycine and hydroxyformylglycine (formylglycine hydrate or a gem-diol) in the active site [7,8,9]. The differences in substrate specificity among the family members are believed to be a result of only subtle differences in active site architecture [2].

ARSB is involved in the degradation of dermatan sulfate (DS) and chondroitin sulfate by hydrolyzing their terminal sulfate residues. Lack or reduced level of ARSB resulting from mutations leads to disruption in the metabolism of these glycosoaminoglycans and their accumulation, and as a consequence to the lysosomal storage disorder known as mucopolysaccharidosis type VI (MPS VI) [10]. DS is considered to be the main storage material [10] because the chondroitin sulfate can be degraded by several other enzymes.

DS is a negatively charged galactosaminoglycan built from disaccharide units containing L-iduronate and N-acetylogalactosamine-4-sulfate (GalNAc-4-sulfate) [11]. Its sulfhydryl and carboxyl groups bind calcium ions, and its affinity for calcium is probably increased by the complex 3D structure of the chain, which depends on specific sulfation patterns [11,12]. In blood and tissues, DS occurs in the form of proteoglycans, which bind and regulate many proteins important for cell functioning, for example, hepatocyte growth/scatter factor, interferon-γ, fibronectin, and thrombin [13]. Interaction of DS with basic fibroblast growth factor (FGF-2) and keratinocyte growth factor (FGF-7) promotes cell proliferation [14,15]. Degradation of DS is a cascade of reactions catalyzed successively by iduronate 2-sulfatase, α-L-iduronidase, ARSB, β-hexosaminidase, and β-glucuronidase. ARSB removes C4 sulphate ester groups from N-acetylgalactosamine at the nonreducing terminus of the DS chain, and disruption of this step stops the process of degradation and leads to accumulation of the GAG in cells and tissues [16]. Clinical features of such storage disorder include short stature, skeletal deformities, pulmonary hypertension, corneal clouding, cardiac abnormalities, and many others [10,17,18].

ARSG is a recently-discovered arylsulfatase presenting high sequence and structure homology with other family members, in particular with ARSA and ARSB: they are all characterized by the very conserved structure of the active site including a modified cysteine with a metal ion in it [3]. So far, ARSG has been shown to catalyze the hydrolysis of pseudosubstrates such as p-nitrocatechol sulfate, 4-methylumbelliferyl sulfate [4], and 3-O-sulfated glucosamine residues of heparan sulfate [5].

We have reported a cellular model of type VI mucopolysaccharidosis (MPS) to investigate these processes at the molecular level using human pulmonary artery endothelial (HPAEC) cells with a silenced *ARSB* gene [19]. In the current work, we expanded this study to cells from the human arterial medial wall. We observed that in pulmonary artery smooth muscle (PASM) cells with a silenced *ARSB* gene, expression of the gene encoding ARSG is significantly upregulated and the proliferation is increased in the presence of exogenous DS. This effect did not occur in HPAEC. Homology modeling of the active site of ARSG and of docking of DS shows plausible geometry for the catalytic reaction, and we therefore propose that ARSG can functionally replace ARSB in cells with a silenced *ARSB* gene.

## 2. Results

### 2.1. Viability of PASM Cells Is Increased by Growth with DS

**Figure 1:**
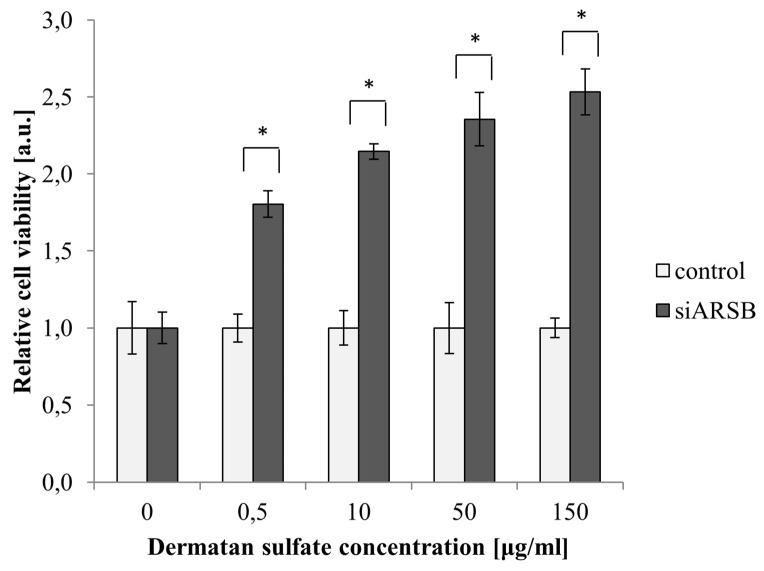
Influence of dermatan sulfate (DS) concentration in media on the viability of pulmonary artery smooth muscle (PASM) cells. In cells with depletion of the arylsulfatase B level (siARSB), increasing concentrations of DS result in stimulation of viability. Results are shown as mean ± SD from three biological experiments. *—statistically significant changes (compared to controls treated with non-targeting siRNA) (*p*-value < 0.05).

PASM cells with a silenced *ARSB* gene presented significantly higher viability than control cells when cultured with DS (Figure 1), indicating that DS stimulates their growth. In contrast, we observed previously that HPAEC cells with a silenced ARSB showed reduced viability in the presence of DS (previously published in [19]), indicating that PASM and HPAEC cells with a decreased ARSB level present different cellular responses in the presence of DS.

### 2.2. Reduced ARSB Expression is Accompanied by Upregulation of ARSG in PASM Cells

No differences that would explain these different responses to DS were revealed by microarray analysis of transcripts coding for enzymes involved in the degradation of DS (Table 1); for both PASM and HPAEC cells treated with siARSB, we observed a change in the level of *ARSB* only. However, analysis of the expression of transcripts for other members of the arylsulfatase gene family (Table 2) revealed that in PASM cells, a reduction of *ARSB* expression was accompanied by the upregulation of *ARSG*, an effect not observed in HPAEC cells.

**Figure 2:**
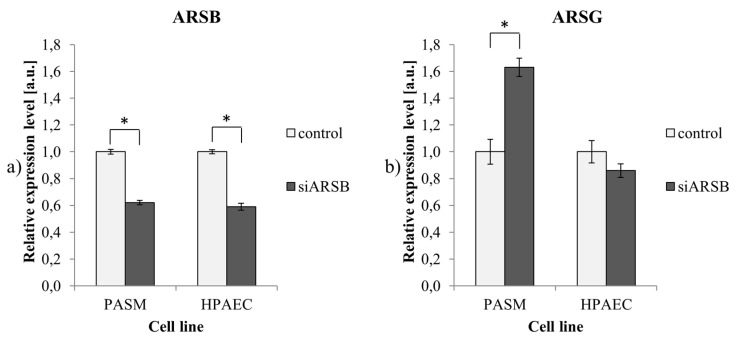
Expression of *ARSB* and ARSG. (**a**) The efficiency of *ARSB* gene silencing in PASM and HPAEC cells using siRNA; (**b**) expression of ARSG transcript in PASM and HPAEC cells with a depleted *ARSB* gene. Results are shown as mean ± SD from three biological experiments. *—statistically significant changes (compared to controls treated with non-targeting siRNA; *p*-value < 0.05).

Validation of these results using RT-qPCR confirmed that the efficiency of *ARSB* silencing was similar in PASM and HPAEC cells (38% and 41% reduction of mRNA level, respectively) and that an increase of *ARSG* expression occurred only in PASM cells (Figure 2).

### 2.3. The Active Site of ARSG is Closely Similar to That of ARSB

**Figure 3:**
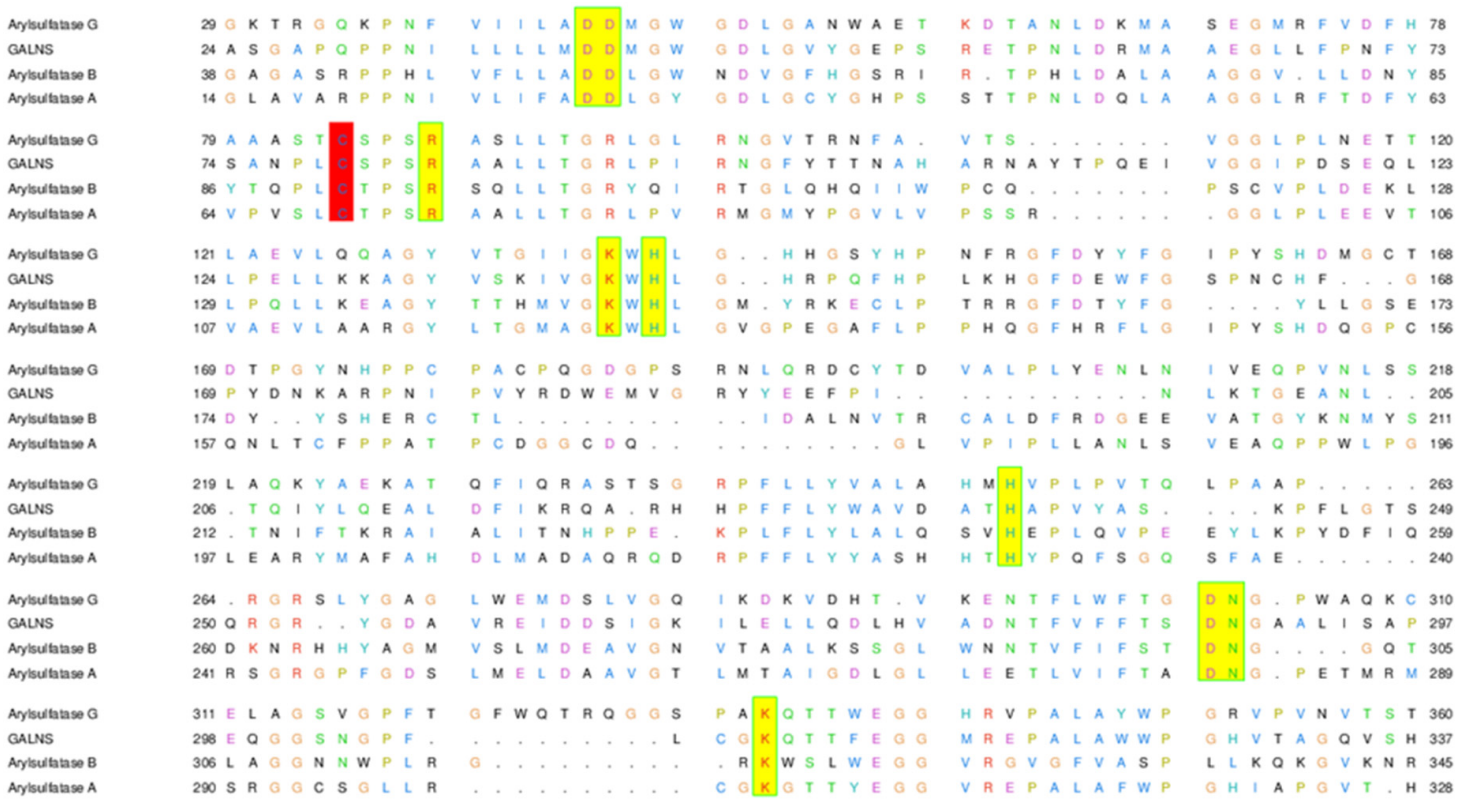
Sequence alignment of ARSG. GALNS template for homology modeling and two representatives of arylsulfatase family ARSB and ARSA. Cysteine modified to formylglycine is marked in red; other conserved amino acids are in yellow.

The upregulation of *ARSG* in PASM cells following a reduction of *ARSB* expression suggested that the ARSG may be competent to replace ARSB, and we therefore compared the molecular architecture of their active sites by molecular modeling. The active site of ARSG is composed of amino acids conserved throughout the arylsulfatase family and present also in ARSB, galactosamine-6-sulfatase (GALNS), and ARSA. Essential amino acid residues are located in almost the same position in all of these enzymes and are listed in Table 3 with the sequence alignment in Figure 3.

**Figure 4:**
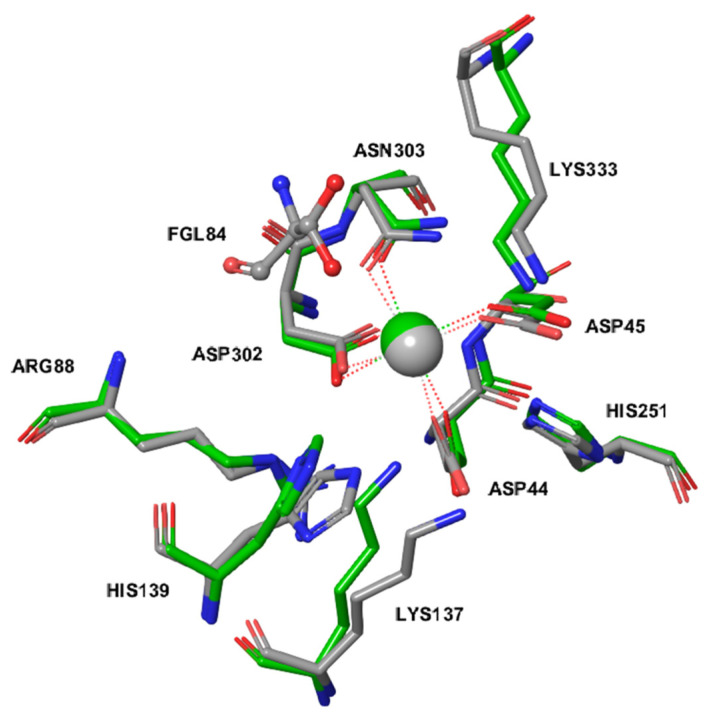
Superposition of catalytically important amino acids in the ARSG homology model (gray) and in ARSB (PDB ID: 1FSU) (green). The formylglycine (fGly)-diol of ARSG is shown in a ball-and-stick representation. Marked in red are oxygen atoms, marked in blue are nitrogen atoms.

Figure 4 shows the superposition of catalytically competent amino acids in the active site of ARSB (PDB ID: 1FSU) and of an ARSG homology model based on a human GALNS template (PDB ID: 4FDI). The active site of ARSG contains all catalytically important residues, and its configuration is very similar to that of ARSB. The active sites contain metal cations modeled as Mg^2+^ ion coordinated by the three aspartate side chains and the asparagine residue.

**Figure 5:**
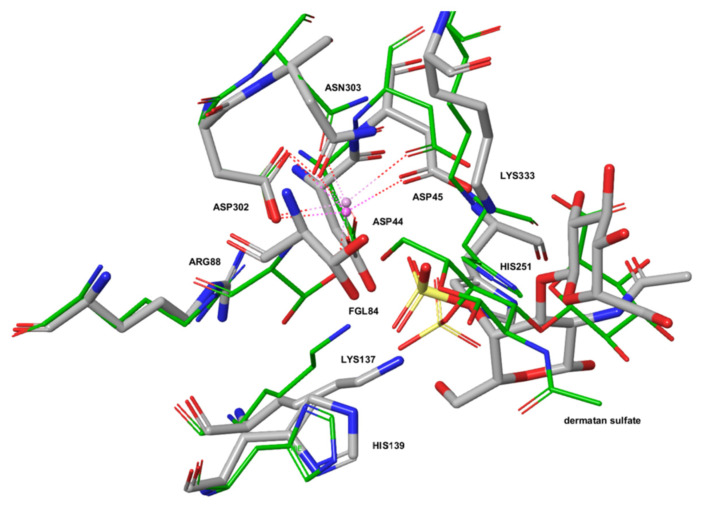
Comparison of binding modes of dermatan sulfate IdoA(a1-3)b-GalNAc4S to ARSB (PDB ID: 1FSU) shown in green and to ARSG (homology model) shown in gray. Marked in red are oxygen atoms, marked in blue are nitrogen atoms.

We compared binding modes of dermatan sulfate IdoA(a1-3)b-GalNAc4S to ARSB and ARSG using the Schrödinger Induced Fit Docking (IFD) protocol. ARSG structure with Cys84 computationally modified to formylglycine (fGly)-diol shows plausible geometry for the catalytic reaction (Figure 5).

## 3. Discussion

Dermatan sulfate (DS) plays an important role in the regulation of migration, proliferation, and synthesis of the extracellular matrix [20]. Disruption of its metabolism leads to its accumulation within lysosomes as well as outside cells, which in turn results in damage to the cells and tissues of almost all organs [16]. One of the diseases associated with disturbances in DS metabolism is mucopolysaccharidosis type VI (MPS VI), caused by a decreased expression and/or activity of ARSB (the enzyme responsible for hydrolysis of the DS terminal sulphate group) [10]. Among the pathological findings in MPS VI is hypertrophy of the medial and intimal layer of arteries due to myointimal proliferation [21,22]. To investigate whether the hypertrophy of blood vessel walls is correlated with the amount of ARSB, we reduced the level of *ARSB* expression with siRNA and then measured cell proliferation using MTS assays. In our previous work [19], we demonstrated that the effect of DS on the proliferation of HPAEC cells with a decreased level of *ARSB* is dependent on DS concentration. At low doses, both DS and DS-SILY20 (a mimic of the proteoglycan decorin consisting of type-I collagen-binding peptides bound to DS) promoted proliferation of endothelial cells [19,23]. However, high concentrations strongly inhibited proliferation, indicating that a decreased ARSB level and the related accumulation of DS in endothelial cells cannot be the direct reason for the narrowing of the blood vessels in MPS VI. Therefore, in the current study, we examined the effect of the ARSB level on the proliferation of vascular smooth muscle cells of pulmonary artery origin (PASM) in the presence of increasing concentrations of DS. DS did not affect the proliferation of PASM cells with the native level of ARSB, in agreement with the results of Scott et al. [24] who found no change in the rate of proliferation of nonstimulated aortic smooth muscle cells cultured in the presence of DS-SILY20. Surprisingly, we found that the proliferation of PASM cells with a reduced level of ARSB depends strongly on the concentration of DS, and that DS significantly increases the rate of division of PASM cells (Figure 1), which suggest that vessel hypertrophy in MPS VI patients could be related to a dysfunction in the smooth muscle cell proliferation ratio rather than in endothelial cells. As Rasente et al. [25] have shown that increased cell proliferation can be induced by low molecular weight DS, our results also suggest that in PASM cells with a reduced level of ARSB, DS may be at least partially hydrolyzed by additional enzymes.

To search for the possible origin of the difference in response to DS between HPAEC and PASM cells with native or reduced ARSB levels, we performed microarray gene expression analysis. We found no significant differences in the expression of genes encoding known enzymes involved in the degradation of DS either in HPAEC or PASM cells (Table 1). Surprisingly, when analyzing the expression level of other representatives of the arylsulfatases family in cells with a reduced level of ARSB, we found that expression of *ARSG* was significantly increased in PASM cells (Table 2). However, there was no increase in HPAEC cells with a reduced level of ARSB.

To investigate whether ARSG could degrade DS, we used molecular modeling methods. The human ARSG active site is composed of amino acids conserved throughout the arylsulfatase family, and its catalytic function depends on posttranslational modification of conserved Cys84 residue to formylglycine (fGly), which in the resting state predominates as fGly-diol (geminal diol) resulting from rapid hydration of fGly. A hydrogen bonding network centered on fGly provides general acid-base catalysis. Additionally, divalent cation (coordinated by glutamine/asparagine), usually Ca2^+^ or Mg2^+^, helps to bind and polarize the substrate coordinated by several acidic residues. The histidine base is predicted to assist in catalysis. A more detailed description of sulfatase catalysis can be found in [26]. Although this general mechanism of catalysis has been originally described for arylsulfatase from Pseudomonas aeruginosa, it is predicted to also be valid for human ARSG given the high conservation of the catalytic amino acid residues and the divalent cation.

The amino acids forming the active site of ARSG are also present in ARSB, GALNS, and ARSA, and the essential amino acids are located in almost the same position in all considered enzymes, suggesting that they should fulfill equivalent roles during catalysis. Our study shows that the configuration of the active sites in ARSB and ARSG is very similar, with only minor conformational differences. Docking of DS to our homology model of human ARSG shows plausible geometry for the catalytic reaction, suggesting that DS can be a substrate for ARSG.

According to our knowledge, this is the first report that shows that ARSG could take part in the degradation of DS and be involved in the regulation of proliferation of smooth muscle cells upon ARSB deficiency.

## 4. Materials and Methods

### 4.1. Cell Culture

Human HPAEC and PASM cells were obtained from Lonza (Basel, Switzerland) and grown in an SmGM-2 medium (Lonza, Basel, Switzerland) with 5% fetal bovine serum or EGM-2 medium (Lonza, Basel, Switzerland) with 13% FBS, respectively, under standard conditions. After plating, cells were transfected with siRNA for 24 h and then incubated with dermatan sulfate (Calbiochem, Sandiego, CA, USA) for another 24 h (concentrations 0–150 µg/mL).

### 4.2. Gene Silencing

The silencing of the ARSB gene was achieved using a siRNA (Thermo Scientific, Waltham, MA, USA) and oligofectamine reagent (Life Technologies, Grand Island, NY, USA) according to the manufacturer’s protocols. A nontargeting siRNA was used as a control. Transfection was performed with a final concentration of 2 nmol siRNA/µl.

### 4.3. Cell Viability

The evaluation of cell viability after the silencing of the ARSB gene and incubation with DS was based on the MTS test using a Cell Titer AQueous Non-Radioactive Cell Proliferation Assay (Promega, Madison, WI, USA) in accordance with the manufacturer’s protocols. Results are presented as mean ± standard deviation. We used Dixon’s Q test for identification and rejection of outliers, and Student’s *t*-test for identification of statistically significant differences (*p*-value < 0.05).

### 4.4. Microarray Data Analysis and Bioinformatics Processing

An RNeasy Mini Kit (Qiagen, Hilden, Germany) was used for purification of high-quality RNA. RNA integrity was determined using the RNA 6000 Nano Kit (Agilent Technologies, Palo Alto, CA, USA). For the microarray experiments, the Agilent SurePrint G3 Human Gene Expression 8x60K (Agilent Technologies, Palo Alto, CA, USA) microarrays were used. Scanning was performed using an Agilent high-resolution G2565CA Microarray scanner and Scan Control 8.5.1 Software to produce two color TIFF images at 532 nm and 635 nm. Raw median signal data were extracted with Agilent Feature Extraction 11.5.1.1 software (Agilent Technologies, Palo Alto, CA, USA). Analyses were performed with the R environment (ver. 3.1.1) and the limma package (ver. 3.24.13) [27] from Bioconductor version 3.1 [28]. Arrays were loess normalized within arrays [29] and quantile normalized between arrays [30]. Agilent positive control probes were removed before normalization. After normalization, negative control probes and probes with all log-intensities below background were filtered from subsequent analysis. Probes were re-annotated using the newest Agilent probe annotation database. Due to the complex probe design, values for within-array replicates were replaced with their average. Values are presented as log2-foldchanges. As significantly differentially expressed genes, we recognized those with a log2FC greater than 0.5 or less than -0.5, and with FDR corrected *p*-values less than 0.05. Results are available in the GEO database under accession number GSE115268.

### 4.5. RT-qPCR

A total RNA Mini Plus kit (A&A Biotechnology, Gdynia, Poland) was used for RNA isolation. Synthesis of the cDNA was performed using Transcriba (A&A Biotechnology, Gdynia, Poland) reagents. The expression level of ARSB and ARSG was examined using RT-qPCR and Realtime PCR Mix EvaGreen (A&A Biotechnology, Gdynia, Poland). Primers sequences were: ARSB forward CTGCCTTTTCACCGTCCTCC, reverse CGCGTCTCCTGTAAAGCCTG; ARSG forward CTAGAAAGAGGTGGTGCGGA, reverse GCAGGGAGTTACTGAAGGGTC. RPL41 was used as a reference to calculate relative expression levels using Livak’s 2−ΔΔCT Method [31] and Student’s *t*-test for the identification of statistically significant differences.

### 4.6. Homology Modeling of Human ARSG

The amino acid sequence of the human ARSG sequence was downloaded from the Uniprot database (entry: Q69EG1) and queried against the Protein Data Bank using BLASTp (with default parameters) to find a template protein homologous to human ARSG. The 3D structure of human galactosamine-6-sulfatase (GALNS) was downloaded from PDB (PDB ID: 4FDI) as the template structure. The homology model of human ARSG was built using Modeller 9.19 (https://salilab.org/modeller/9.19/release.html). The target human ARSG and template human GALNS were aligned with the salign algorithm in Modeller. The calcium ion included in the template structure was retained while other ligands were removed before homology modeling. The 3D structure of the homology model of human ARSG was prepared using the Protein Preparation Wizard in Maestro 11 (Schrödinger, New York, NY, USA). Conserved Cys84 was computationally modified to fGly-diol (formylglicyne-diol) using 3D Builder in Maestro 11. The protein was atom typed and protonated, bond orders were assigned, and disulfide bridges were created. Subsequently, the hydrogen bonding network was optimized using PROPKA (pH 7.4) (Schrödinger, New York, NY, USA). The final structure was subjected to restrained minimization in the OPLS3 force-field [32].

### 4.7. Ligand Docking

The 3D structure of dermatan sulfate IdoA(a1-3)b-GalNAc4S was prepared with Ligprep (Schrödinger, New York, NY, USA) with the OPLS3 force field. The ionization state was determined at pH 7.4 using Epik (Schrödinger, New York, NY, USA). The optimized 3D structure was docked to the homology model of human ARSG and crystal structure of human ARSB (PDB ID: 1FSU) using the Schrödinger Induced Fit Docking (IFD) protocol (Schrödinger, New York, NY USA) with standard settings (Glide eXtra Precision in the redocking stage). The grid center was set as the centroid of residues Gly137, Thr139, Phe162, and Tyr251, forming the ARSG substrate binding site. The best IFD docking result of DS to ARSG was selected according to the IFDScore parameter and, additionally, similarity to pose of DS in ARSB structure (in terms of RMSD measure), as several poses with an almost identical IFDScore were obtained.

## 5. Conclusions

We investigated the effects of decreased arylsulfatase B gene expression in different types of cells of pulmonary artery origins. We found that endothelial and smooth muscle cells presented different responses, especially when cultured in the presence of dermatan sulfate (arylsulfatase B substrate). The proliferation of pulmonary smooth muscle cells was significantly increased, despite the lower level of arylsulfatase B, which should lead to an accumulation of dermatan sulfate. Such an effect has not been observed in pulmonary artery endothelial cells. We used a global approach and performed microarray analysis to find genes associated with these differences in cellular response. There were no changes in expression of genes involved in dermatan sulfate degradation; however, we discovered that the level of one of the arylsulfatases (ARSG) was altered in smooth muscle and not in endothelial cells. ARSG belongs to the same family as ARSB (and both enzymes are localized in lysosomes). However, its function has not yet been fully understood, and it has never been linked to dermatan sulfate metabolism.

We performed in silico analysis of ARSG structure, proving that the active sites of both enzymes (ARSB and ARSG) are highly similar. Molecular docking of dermatan sulfate IdoA(a1-3)b-GalNAc4S to ARSG suggests possible enzyme–substrate interaction. The biological consequence of such a reaction requires further investigation; however, we believe that this finding may be an important step on the way to a clarification of the pathogenesis of hypertension and other cardiovascular diseases in patients with mucopolisaccharidosis type IV.

## Figures and Tables

**Table 1:** Expression of mRNAs of glycosaminoglycan (GAG)-degrading enzymes involved in DS metabolism in human pulmonary artery endothelial (HPAEC) and PASM cells upon ARSB gene silencing. Expression of arylsulfatase B (*ARSB*) was decreased in both HPAEC and PASM cells with similar efficiency. Data from microarrays experiments are shown as the change between expression in cells transfected with siARSB and non-targeting siRNA. Genes with significantly different expression are marked with an asterisk.

Gene	Expression (log_2_FC)	
	HPAEC	PASM
Enzymes Involved in Dermatan Sulfate Metabolism		
Iduronate 2-sulfatase	0.18	−0.13
A-L-iduronidase	0.11	−0.03
Arylsulfatase B	−1.78 *	−1.63 *
β-Hexoaminidase A	−0.15	0.04
β-Hexoaminidase B	0.05	−0.05
β-glucuronidase	0.09	−0.04

**Table 2:** Expressions of arylsulfatases transcripts in HPAEC and PASM cells with decreased expression of the *ARSB* gene. In PASM cells, reduction of *ARSB* expression is accompanied by upregulation of arylsulfatase G (*ARSG*), but this effect is not observed in HPAEC cells. Data from microarrays experiments are shown as the change between expression in cells transfected with siARSB and non-targeting siRNA. Genes with significantly different expression are marked with an asterisk.

Gene	Expression [log_2_FC]	
	HPAEC	PASM
Arylsulfatases		
Arylsulfatase A	0.08	−0.10
Arylsulfatase B	−1.78 *	−1.63 *
Arylsulfatase D	−0.22	0.04
Arylsulfatase E	0.18	−0.23
Arylsulfatase F	0.22	0.08
Arylsulfatase G	0.004	0.67 *
Arylsulfatase H	−0.08	−0.03
Arylsulfatase I	−0.02	0.06
Arylsulfatase J	−0.43	−0.02
Arylsulfatase K	0.12	−0.12

**Table 3:** Amino acid residues are located in almost the same position in ARSG, ARSB, arylsulfatase A (ARSA), and galactosamine-6-sulfatase (GALNS) and are expected to play equivalent roles in catalysis.

ARSG	ARSB	GALNS	ARSA
Asp44	Asp53	Asp39	Asp29
Asp45	Asp54	Asp40	Asp30
FGly84	FGly91	FGly79	FGly69
Arg88	Arg95	Arg83	Arg73
Lys137	Lys145	Lys140	Lys123
His139	His147	His142	His125
His251	His242	His236	His229
Asp302	Asp300	Asp288	Asp281
Asn303	Asn301	Asn289	Asn282
Lys333	Lys318	Lys310	Lys302

## Abbreviations

ARSA	Arylsulfatase A
ARSB	Arylsulfatase B
ARSG	Arylsulfatase G
LD	linear dichroism
DS	Dermatan sulfate
FGF-2	Fibroblast growth factor
FGF-7	Keratinocyte growth factor
fGly	Formylglycine
GAG	Glycsaminoglycans
GALNS	Galactosamine-6-sulfatase
HPAEC	Human pulmonary artery endothelial cells
MPS	mucopolisaccharidosis
PASM	Human pulmonary artery smooth muscle cells
